# A qualitative understanding of the effects of reusable sanitary pads and puberty education: implications for future research and practice

**DOI:** 10.1186/s12978-017-0339-9

**Published:** 2017-06-27

**Authors:** Julie Hennegan, Catherine Dolan, Laurel Steinfield, Paul Montgomery

**Affiliations:** 1 Centre for Evidence-Based Intervention, University of Oxford, 32 Wellington Square, Oxford, OX1 2ER UK; 2 SOAS, University of London, London, UK; 3 Bentley University, Waltham, MA USA; 4 Department of Social Policy and Social Work, University of Birmingham, Birmingham, UK

**Keywords:** Menstrual hygiene, Menstrual health, Adolescent girls, Education, Qualitative

## Abstract

**Background:**

The management of menstruation has come to the fore as a barrier to girls’ education attainment in low income contexts. Interventions have been proposed and piloted, but the emerging nature of the field means limited evidence is available to understand their pathways of effect.

**Methods:**

This study describes and compares schoolgirls’ experiences of menstruation in rural Uganda at the conclusion of a controlled trial of puberty education and sanitary pad provision to elucidate pathways of effect in the interventions. Semi-structured interviews were undertaken with schoolgirls who participated in the Menstruation and the Cycle of Poverty trial concurrent with the final set of quantitative surveys. A framework approach and cross-case analysis were employed to describe and compare the experiences of 27 menstruating girls across the four intervention conditions; education (*n* = 8), reusable sanitary pads (*n* = 8), education with reusable sanitary pads (*n* = 6), and control (*n* = 5).

**Results:**

Themes included: menstrual hygiene, soiling, irritation and infection, physical experience, knowledge of menstruation, psychological, social and cultural factors, and support from others. Those receiving reusable pads experienced improvements in comfort and reliability. This translated into reduced fears around garment soiling and related school absenteeism. Other menstrual hygiene challenges of washing, drying and privacy remained prominent. Puberty education improved girls’ confidence to discuss menstruation and prompted additional support from teachers and peers.

**Conclusions:**

Findings have important implications for the development and evaluation of future interventions. Results suggest the provision of menstrual absorbents addresses one core barrier to menstrual health, but that interventions addressing broader needs such as privacy may improve effectiveness. Puberty education sessions should increase attention to body awareness and include strategies to address a wider range of practical menstrual challenges, including pain management. Interviews revealed possibilities for improving quantitative surveys in future research.

**Trial registration:**

Pan-African Clinical Trials Registry PACTR201503001044408

## Plain English summary

The effective management of menstruation has been identified as an under-recognised challenge for girls in low income contexts. In response, there has been increasing emphasis on the potential for interventions addressing this issue to improve girls’ health, education and psychosocial wellbeing. This paper follows up a controlled trial testing the effectiveness of providing girls in rural Uganda with puberty education or reusable sanitary pads to improve their school attendance. The trial was undertaken in an area characterised by poor education, health and welfare, and found positive results for both interventions on attendance, but not on psychosocial outcomes such as shame and insecurity. This study uses in-depth interviews with 27 menstruating girls from the trial to better understand how the interventions worked. We describe and compare girls’ experiences of menstruation across the four trial conditions (education, reusable sanitary pads, education with reusable sanitary pads, and control). We found that menstruation presented a significant challenge to school attendance, and that the provision of sanitary pads reduced fears of soiling outer garments allowing better school attendance. Providing pads did not address other challenges of managing menstruation such as washing and drying the reusable pads. The education intervention was helpful in promoting understanding of menstruation, but more detailed information and practical strategies may improve future education packages. Across the interventions we found social support from peers and teachers to be an unmeasured driver of intervention effects. Finally, we found that menstrual pain and variations in menstrual experience cause significant distress and suggest some strategies for interventions to address this issue.

## Background

Many challenges lie ahead in achieving quality education for all. Investment has seen considerable improvements in education metrics across low and middle income countries (LMICs). However, progress has been uneven and poor girls remain the most disadvantaged, facing an array of barriers to school enrolment, attendance, and attainment [1]. Among these, menstruation has emerged as a neglected but significant obstacle to education.

Although an individual biological event, the experience of menstruation is entangled within a complex socio-ecological system. Reflecting this, ‘*menstrual health’* has been suggested as an overarching term for women and girls’ menstrual experience encompassing menstrual hygiene, the physical requirements of effective management, as well as broader psychological and socio-cultural elements [2]. Menstrual health is severely under-researched. Consequences of inadequate menstrual management have been suggested to extend beyond the classroom to health, dignity, psychosocial wellbeing, employment, and participation in society [3, 4]. Nuanced understanding of this complex experience is needed in the development of interventions, and in their evaluation [5, 6]. Programs seeking to improve menstrual health have been implemented, but few high quality trials have been conducted [7].

Across disciplines there is increasing recognition of the value of qualitative research conducted alongside effectiveness trials [8, 9]. Lewin and colleagues [8]. suggest four broad ways qualitative methods can be employed after a trial to enhance evaluation: to explore reasons for the findings; to explain variations in effectiveness across the sample; to examine underlying theory, and; to generate further questions or hypotheses. Furthermore, in fulfilling these functions qualitative research serves to provide a trial process evaluation by exploring mechanisms of impact in the trial, implementation, and study context [10]. This is highly appropriate in the study of menstruation, where evidence is sparse and interventions are undergoing rapid development and modification.

The present study uses this qualitative approach to describe and compare experiences of menstruation at the conclusion of a controlled trial of reusable sanitary pad and puberty education provision in rural Uganda [11]. By comparing girls’ experiences across trial conditions this work aims to elucidate pathways of effect in the interventions and to identify barriers and facilitators of intervention effectiveness and implementation. In this way, the study enhances the quantitative trial evaluation and provides recommendations for future intervention development and evaluations.

### The menstruation & the cycle of poverty trial

This study follows-up the *Menstruation and the Cycle of Poverty* trial; a quasi-randomised controlled trial of the impact of reusable sanitary pad and/or puberty education provision on school attendance amongst girls in rural Uganda. The methods and results have been reported elsewhere [11]. In brief, eight schools in Kamuli District, located in areas characterised by poor educational attainment and high rates of school drop-out were assigned to one of four conditions. Schools were grouped into pairs based on geographical proximity and assigned alphabetically. Each of the pairs received either puberty education (hereinafter referred to as education) alone, reusable sanitary pads alone, both pads and education, or no-intervention (control). The education comprised a 75-min session providing information about puberty and managing menstruation. The Straight Talk education program was selected for its high face-validity and emerging evidence base [11], (full program available online www.spi.ox.ac.uk/fileadmin/documents/PDF/Straight_Talk_Plan.pdf). Reusable sanitary pads were AFRIpads, a locally produced cloth pad, which were provided with a single sachet (45 g) of Omo soap, three pairs of underwear, and instructions on how to use and clean the pads. AFRIpads were distributed in kits which included two plastic-lined ‘base’ pads, three attachable winged liners, three straight liners and washable carry bag. AFRIpads were selected following an acceptability study reported elsewhere (see [12]).

The trial found that the percentage of school days attended dropped substantially in the control schools. This drop was mitigated by a mean 17.1% (95%CI 8.7–25.5) by the provision of either education or reusable pads in the intervention schools [11]. There were no significant differences in attendance between the intervention conditions, nor between psychosocial wellbeing outcomes, including shame and insecurity during menstruation, when compared at the conclusion of the trial.

## Methods

### Data collection

Twenty-seven girls were interviewed in October 2014 concurrent with the final quantitative survey data collection in the *Menstruation and the Cycle of Poverty* trial. Girls were eligible for interview if they had been at the school for longer than 2 years (since the start of the trial), and had reached menarche. Participants were sampled at each of the eight study schools and selected from a range of school grades (primary classes 4–7) and ages. Participants were aged 12 to 17 (*Mean* = 14.35, *SD =* 1.11) and the time since menarche varied. Interviews were spread between the four intervention conditions of education alone (*n* = 8), pads alone (*n* = 8), education and pads (*n* = 6), and control (*n* = 5).

Members of the research team (JH, LS) interviewed girls over approximately one hour at a private location on the school grounds. Lusoga translations were provided by trained female research assistants from the partner NGO. Interviews were digitally recorded. Both English and Lusoga portions were transcribed.

Semi-structured interviews covered a range of topics drawn from interviews undertaken in pilots preceding the trial, [12, 13] with additions to capture more detail around menstrual experience and trial implementation (see Table 1 for broad guide). Following the topic guide, girls were asked open-ended questions about; their family circumstances, schooling, experiences of menstruation and menstrual management, perceptions of other girls’ menstrual management and the impact of the trial conditions, awareness of reproductive health and contraception, and their future ambitions. To address the research questions of this study, transcripts were coded for menstrual experience and trial implementation. Broader investigation of sociocultural gender norms, family dynamics and education expectation aspects of the interviews were undertaken separately.

**Table 1:** Broad interview topic-guide

Broad interview topic-guide	
-Household dynamics	
-Schooling history	
-Payment of school fees and access to resources	
-Attitudes/expectations of education and gender	
-Menstrual experiences	
-Menstrual practices	
-Perceptions of menstruation at school and other girls’ experiences	
-Sources of menstrual information/support	
-Knowledge of family planning	
-Future ambitions	

In addition to interview transcripts, notes from school facility assessments, field notes and debrief sessions with the project research assistants were consulted to contextualise interviews. School facility appraisal included the number and condition of the latrines, distance to water, access to water and soap, and school fees and grain requirements.

### Analysis

Analyses were conducted using NVivo 11. Interview transcripts were analysed using a framework approach with cross-case analysis to identify differences in experiences between intervention conditions. Framework analysis employs a thematic analytic approach with structured data management for a comprehensive, systematic analysis and data summary. The technique is well suited to large quantities of data, and studies with pre-established research questions.

Framework analysis followed the five steps described by Spencer and colleagues [14].

1. Familiarisation: Four interviews were transcribed by the analyst (JH) and a further three read in their entirety.
2. Initial thematic framework: A blended approach of deductive and inductive coding was used. The initial thematic framework was developed based on past literature and the quantitative findings from the trial. This was updated with emerging themes identified in familiarisation, indexing, and sorting (step 3). Final thematic framework is displayed in Table 2. Few changes were made. Privacy was initially listed as a subtheme of menstrual hygiene, but was removed as it was an inextricable part of other aspects of menstrual hygiene (e.g., drying).
3. Indexing and sorting: All interviews were coded into the thematic framework, with emerging themes added as described above.
4. Reviewing data extracts: Theme and sub-theme coding was reviewed and discussed among the study authors (JH CD LS PM).
5. Data summary and display: A framework matrix was generated using NVivo and each cell filled with a written summary and illustrative quotes by the first author.

**Table 2:** Final thematic framework

Final thematic framework	
Theme	Sub-theme
Menstrual hygiene	Absorbent
	Changing absorbents
	Washing
	Drying absorbents
	Disposal
Soiling	Soiling
	Absorbent falling out
Irritation & infection	
Physical experience	Menstrual symptoms & management
	Individual menstrual factors
Knowledge of menstruation	
Psychological, social and cultural factors	Coping mechanisms & self-efficacy
	Stigma & shame
	Labia elongation
Support	Teachers
	Friends
	Family
	Others

Following the framework analysis, cross-case matrices were generated to compare between the trial conditions.

## Results

Findings are presented using the framework themes (Table 2) with differences between conditions discussed within each section.

### Menstrual hygiene

#### Absorbents

Cloth was the most common absorbent for girls who did not receive AFRIpads. Some girls used clean, new cloths such as face towels or large sheets cut into pieces. Such cloths were purchased by or with funds from caregivers. Other girls used their own wages from farming work to purchase cloths or disposable pads. Those who could not afford cloth or pads used found-cloth such as old sweaters or discarded rags. In some cases, girls used underwear alone or multiple pairs of underwear. While girls were usually aware of commercial pads and may have used them, most could not afford them. A few girls reported that parents had purchased pads, but most did so inconsistently.

Unreliable absorbents kept girls from school, particularly where girls were using old or found cloths. Girls with no absorbents were absent. Most girls reported knowing others who struggled to find absorbents and thus stayed home.

*“That like now, I began 3 days ago, but I did not come to school those three days, and I have just come back today.” (Participant 16, Pads-only)*

*Participant: “I stay home on the day I start.... Am not ready by that time but the following day I tell mummy to buy me pads....”*

*Interviewer: “What do you use the first day?”*

*Participant: “I use a cotton cloth.”*

*Interviewer: “Why don’t you come to school while using that cloth?”*

*Participant: “I fear that it may leak out.” (Participant 25, Control)*

Intervention education sessions taught girls to sew pads from cloth. Girls reported trying to do so, but felt unable to sew the pads properly or lacked resources such as sewing equipment. At one education-alone school girls had more success, forming a group to pool resources and purchase large sheets of fabric and sewing needles to make pads as a joint venture. Parents were supportive, sometimes providing the fabric sheets. These were given to girls in need, or sold to those who could not make their own.

*“Those who don’t have admire us and buy from us, others take it to be very important and they want us to be very important and they want us to teach them of which we do.” “…Even hygiene has improved at school. Other girls where using these papers to clean and put in the toilet, you could find the whole toilet dirty. They could use the other leaves, those long ones you have seen. They could use those leaves to clean themselves even plastic papers, so the cleanliness of the toilet was bad, but since we started giving out pads people can contain until they go home...” (Participant 8, Education-only)*

Girls receiving AFRIpads felt they were superior to previous improvised absorbents in comfort and reliability. When using various types of cloth, girls often reported discomfort from wearing multiple pairs of underwear, whereas fewer pairs of underwear were worn when using the AFRIpads. Providing AFRIpads also reduced financial burden on girls. A participant from a pads-only school expressed comfort with the AFRIpads and reported that previously *“I just used cloths, but later found out that I couldn’t manage them so I started working for people to buy pads”* (Participant 12). There were some concerns raised about AFRIpads: a few girls reported that they experienced a burning sensation, or heard that others experienced this, while other girls were concerned about the pad moving or being visible.

*“…when you put it on, sometimes when you are walking it keeps on moving that way and backwards. They can protrude behind or in front.” (Participant 15, Pads-only)*

#### Changing absorbents

The type of absorbent used, particularly the amount of cloth, impacted changing practices. For some, having few cloths meant that when one was removed it needed to be washed and dried immediately so that it was dry in time to change again. Since no school had washing facilities, this required going home.

*“... I put on in the morning, I go back during lunch time and I change and even wash again at 4:00pm. The others may be dry and I go and wash, and that is what I put on at night and put the one I washed on in the morning, and again wash the one I used at night” (Participant 23, Control)*

For those with sufficient absorbents to allow for changing at school, practices varied across the schools despite comparable latrines. At one school (education-only), a common bullying practice was to lock other students inside the latrines, compelling all girls interviewed at this school to return home to change. Conversely, at another school (pads-only condition) girls regularly changed absorbents in the latrines, storing used AFRIpads or cloth in a black plastic bag placed into their schoolbag to take home and wash. Going home to change was most common among those in education-only and control schools. Those living close to the school may return after changing, others stay home.

*“I go back home at 10am or 1pm and change… I normally come back. But I might spend 45 minutes because it is far.” (Participant 7, Education-only)*

In one school (education-alone), girls reported changing, and sometimes washing, absorbents at the home of a female teacher living close to the school, allowing them to return to school quickly after changing.

#### Washing, drying and disposal

Single-use pads were disposed of in school pit latrines. Girls using AFRIpads or cloth washed, dried and reused them. Two girls who boarded at school washed absorbents in the evening and hid them to dry in the girls’ dormitory. Most girls had access to soap, although for some this was inconsistent. Girls developed different strategies for accessing soap including purchasing it themselves, borrowing money, or using a local plant known to act like soap.

*“When I do not get soap sometimes I go to a boy who knows us and we also know him and tell him to give me soap that if I get money I will give you”… “When I borrow I go and get a portion and dig for money and pay back” (Participant 23, Control)*

Girls in pad-provision conditions used the small quantity of Omo soap provided surprisingly sparingly, possibly stretching the 45-g over nine months in some cases.

*“I use omo detergent to clean the pad. When it gets finished I can ask for money to pay for more.” (Participant 9, Pads-only)*

When girls lacked soap most would discard the used cloths.

*“I throw them away if mother has no soap. I throw them away since I can’t keep dirty things and do not want people to see dirty things...” (Participant 24, Control)*

Although girls expressed comfort with having water and their own basin or female-only bathroom area for washing cloth or AFRIpads at home, there were privacy concerns around drying absorbents.

*“… if there are no people you can quickly put it outside to dry.” (Participant 4, Education-only)*

This did not differ between those using cloth, and those using AFRIpads. When drying absorbents outside girls hid them in bushes, put them on top of poultry houses, or hung them on wires. The absorbent was then covered with another thin piece of material to conceal it, a practice reported by some girls drying inside in more private places such as shared bedrooms as well. Girls sharing their bedroom with only females hung absorbents on a nail or wire in the room. For girls boarding at school, there were concerns around boys seeing pads drying, while those drying at home sometimes reported hiding them from parents.

### Soiling

All girls expressed fears of soiling. Girls’ who received AFRIpads felt these were reliable and experienced greater comfort as a result, with some reporting that they experienced soiling prior to, but not after, receiving AFRIpads. Soiling was a consistent problem for girls with poorer quality absorbents such as old cloth or underwear alone, prompting some to stay home on the heavier days of their period. All girls recalled others soiling at school, and expressed compassion for girls they knew soiled frequently. Fear of soiling often meant girls avoided standing in class to answer questions, and was a barrier to concentration.

*“…there is one girl and she experiences leaking almost every month. She gets teased for it, mostly by the boys.” (Participant 9, Pads-only)*

*“…you cannot settle because you really feel blood is going to soil your uniform any time.” (Participant 23, Control)*

One participant was the ‘head girl’ for her class meaning that others came to her if they had soiled their outer garments. She reported that this happens monthly. Fears about soiling were amplified by girls’ concern that absorbents, particularly cloth, would fall out of their underwear. Girls described sitting with their legs pressed together in class, and not playing games or skipping to avoid embarrassment.

Despite girls’ focus on soiling through absorbents, most reported that soiling at school occurred on the first day of their menses when they were unprepared. One girl stated that she no longer experienced soiling since receiving AFRIpads, but still might be caught unaware by her period. At one school the uniforms had changed colour in the past year, reducing girls fears of soiling.

*“because you may come to school when you are ok and it starts at school then you say “ha” since it has just started it will not come out. You just see at 10am when you stand it has spilled.” (Participant 4, Education-only)*

*“… this type of uniform has helped us a lot because it is heavy and the colour is not bad. But before when we were in yellow, you could even see someone in class when the uniform is full of blood. But now it is better because we are in this uniform.” (Participant 8, Education-only)*

### Irritation and infection

Genital irritation was reported across the whole sample. Some girls’ described a burning sensation when using the AFRIpads, associating it with wearing the pad for an extended time. Irrespective of absorbent type, girls described how they were unable to deal with discomfort such as irritation during class, and needed to wait to get away from the classroom or school during a break.

*“That sometimes, this begins when the teacher has entered in class. You need to finish the lesson, if you go out you will not get what the teacher has told, it will keep on burning until the teacher goes out.” (Participant 6, Education only)*

Many girls, across the conditions, had experienced itching and abnormal discharge at least once. They did not have an explanation for why this occurred, nor did they tell or ask anyone about it. Many were embarrassed, or felt that care providers might share the information with others.

### Physical experience

#### Menstrual pain

Menstrual cramping was a focal part of girls’ experience across all conditions. They reported school absences and disengagement for themselves and others due to pain. Participants often named two or more girls from their class who experienced significant pain and stayed home.

*“I worry a lot about periods because I have serious cramps and the back pains a lot.” (Participant 20, Pads & Education)*

*“…if they are too much, I don’t come and if they are not too much I come.” (Participant 12, Pads-only)*

Pain management practices were mixed. Some girls had never taken steps to reduce their pain, while others frequently used paracetamol. Girls’ sought to access painkillers through their care providers (without revealing menstruation as the source of pain) or purchased them with funds from their labour. Care providers sometimes provided painkillers. Paracetamol was the most common product, although some girls did not know what tablets they were given. One girl sought help from a health clinic, but only told them that she had stomach pain and was sold an anti-malarial. Girls reported mixed effectiveness of painkillers.

#### Individual menstrual factors

Girls expressed anxiety about variations in their menstrual patterns, which they feared were abnormal. This was most the common concern girls raised when they were provided the opportunity to ask questions of the interviewer. This was similar across all conditions, and did not seem to be affected by having received the education intervention. In particular, girls were concerned that their bleeding was too heavy, although it was unclear to interviewers if bleeding was truly abnormal.

*“I menstruate for a full week, can you help me out?... In the first five days, it is heavy, but light in the last two.” (Participant 12, Pads-only)*

One girl reported month-long bleeding, and another reported instances where her menses had come two weeks apart. In these cases, girls feared that something was very wrong with them. Girls had sometimes sought advice from female teachers or relatives. Advice received was limited, with others simply encouraging girls that they were normal. Symptomatology suggested possible menstrual disorders in a couple of cases.

### Knowledge of menstruation

Regardless of intervention condition, many girls had known about menstruation prior to menarche, although were given varying information by mothers, aunts, sisters, or peers. In some cases, girls reported that teachers had first informed them, or had provided missing details. Teacher-provided sessions on menstruation were reported in some control and pad-only schools; some appeared to be special sessions arranged by female teachers, while others were part of classes on reproduction. For a couple of girls, the intervention was the first time they had learned about menstruation. Exposure to the education prompted some girls to start discussions with caregivers about menstruation. Girls in education conditions were also more comfortable discussing menstruation in interviews. Receiving the pads-alone intervention did not appear to have the same impact, and notably two girls from the pad-only schools expressed fear to describe menstruation or use anatomical terms such as ‘vagina’.

Despite being aware of menstruation, many girls reflected on menarche with fear. Although they had been told that menstruation would happen to them, most received little instruction on practical management. A number of girls reported that the intervention education session was the first time they received any demonstration of the use of absorbents. Education sessions provided girls with more practical knowledge than they had before, such as the importance of using cold water for washing. One girl reflected that prior to the education she *“didn’t know how to put the pads in the knickers very well” (Participant 19, Pads & Education).* In some instances, girls were told by family members to restrict their activities during menstruation, including not going to school or being around boys. The education sessions informed them otherwise. Disparity between the advice provided by family members or peers and information in the education sessions was a source of stress. This is noted below with regard to practising labia pulling, but was also evident in other decisions such as whether to attend school or engage in other physical activities.

### Psychological, social and cultural factors

Anxiety and embarrassment pervaded menstrual experiences across conditions. Girls carried significant anxiety about soiling outer-garments and being embarrassed or teased.

*“That mostly when it soils they start bullying you. Both girls and boys.” (Participant 20, Pads & Education)*

*“Most of the girls they feel shy when they are in their MPs [menstrual periods].” (Participant 8, Education-only)*

This anxiety was reflected in girls’ recollections of menarche. Even though they had been told that menstruation would happen, most did not immediately tell care providers or friends about it. Many tried to manage on their own, and waited until carers noticed and asked them about it.

As noted above, the AFRIpads allowed girls to feel more confident that they would not soil garments during menses and girls receiving education more freely discussed menstruation with the interviewers. This confidence appeared to have extended to interactions with others. Girls in the intervention schools reported greater ease discussing menstruation with peers, albeit with some individual variation. This contrasts with girls in the control schools, two of whom stated that they could not talk to friends about their menses at all.

Across intervention conditions, girls were heavily reliant on external sources to tell them what was normal for their periods and what their management practises should look like. Girls’ reliance on others to provide funds for absorbents, soap and basins for washing meant most expressed little control over their ability to manage menses. Furthermore, girls expressed little control over their body. Of notable exception were the few girls whose parents regularly provided disposable pads and funds for painkillers. These girls expressed greater control and lower anxiety.

### Labia elongation

Labia elongation emerged as an important cultural practice and avenue through which girls were informed about menstruation. Girls had received instructions from mothers, aunts, teachers and other girls to *‘pull’* and elongate their labia minora. Debriefing sessions with the local research assistants revealed that girls were expected to elongate labia prior to menarche at which time labia were believed to become tougher and harder to elongate. The practice came to light in interviews when girls were asked who had told them about menstruation, with girls being told by teachers (male and female alike) in reproductive classes to pull their labia, or when girls were provided the opportunity to ask questions of the interviewer. Girls were conflicted about whether or not they should engage in the practice, and indicated that the topic had been raised during questions in the education sessions.

*“They [teachers] told us that in case you didn’t pull the labias, you should pull, and in case you see blood from the vagina do not fear for it is normal” (Participant 26, Control)*

*Participant: “Our senior woman teacher advised us to pull the labia but when Plan [the partner NGO] came they told us it is nonsense they should not do that.”*

*Interviewer: “What do you think about who is right, Plan or the senior woman teacher?”*

*Participant: “The teacher who heads girls. Because even at home, mother told us the same.” (Participant 4, Education-only)*

### Support

#### Teachers

Female teachers were an important source of information and support. Girls often felt they could talk to a female teacher about menstrual issues. Notably, the range of topics and the ease of talking to the female teacher was greater in the intervention conditions than the control, where menstrual education at the school was more limited. While most girls reported positive experiences with teachers, some expressed that teachers of both sexes were not sensitive enough to menstrual issues.

*“I was ashamed so I had to leave class and went home, but by that time when I was in class. Madam asked for me and I tried to explain, madam couldn’t believe me so I cried again to explain to her in order to believe me.” (Participant 25, Control)*

At both education-alone schools, girls felt cared for by a female teacher. At one school a teacher provided a safe place for changing absorbents at her house nearby, while at another, the teacher provided spare skirts for girls if they had soiled, and took action to dismiss the boys from class first. There were also reports in other intervention schools that teachers provided girls with skirts in case of soiling.

*“I tell my friend who is next to me who goes and tell the head girl to go to the madam [senior female teacher], tells her and she gives me a skirt which resembles my uniform and I put on then go home.”... “if boys are in madam comes and tells the head girl to send boys out, then she brings the skirt.” (Participant 4, Education-only)*

#### Friends

Across all schools, girls reported checking each other’s skirts for leaks, talking about menstruation with others, and providing moral support. There were a few exceptions of girls who did not feel they could trust others with any information about their menses or feared teasing from other girls, interestingly both of these were in the control schools. Teasing was mostly attributed to boys. One girl in a control school reported that since her father provided pads for her she would sometimes share them with her friends. No girls reported sharing the AFRIpads provided in the intervention.

*“A teacher might be teaching and then a friend stands up when she has soiled her clothes, you advise her to go out she leads the way and you follow to shadow her up. You get her other clothes to put on” (Participant 22, Pads & Education)*

#### Family and others

Mother’s had often told girls about menstruation and provided money for cloth or painkillers. This was consistent across conditions. Where girls did not feel comfortable to discuss menstruation with their mother there was often an aunt, grandmother, or sister they talked to. This varied according to girls’ family circumstances such as living arrangements, the presence of other wives and children, and how much time they spent with their mother. The level of detail in information girls’ received from family varied. For some, they had been told to *“keep clean”* but were not given any formal instruction on absorbent use. Some girls reported copying the use of cloth in their underwear from mothers or sisters. In a few cases, aunts had warned against attending school or collecting water while menstruating. Most girls did not talk to fathers about menstruation. There were a couple of exceptions where fathers were the primary care-giver and girls reported that they could ask him directly for help.

## Discussion

This qualitative follow-up to the *Menstruation and the Cycle of Poverty* trial extends on the quantitative findings, provides a more nuanced picture of the pathways of effect, and illuminates new considerations for menstrual health interventions and research.

### Main findings

Interviews confirmed that among this sample, inadequate absorbents were a source of school absence. This was consistent with past qualitative work (e.g., [3, 15, 16]). The provision of reliable absorbents in the intervention addressed soiling and fears of soiling, and meant girls could more easily attend school during menses. This may have been particularly beneficial for the most disadvantaged girls who struggled to access sufficient clean cloth to use as absorbent. Further, the provision of reusable pads meant that girls could direct precious funds spent on pads or cloths to other needs, a potential added benefit. The ingenuity of the girls in one school to pool resources and make pads is testament to the importance of this issue in their lives and likely contributed to the education intervention effectiveness identified in this school. Beyond this group, no other girls sewed their own pads. Other content in the education sessions such as practical suggestions about how to fix absorbents in underwear or use cold water for washing was beneficial for girls’ menstrual management.

None of the interventions addressed the school facilities available for changing absorbents, which were similar across the schools. School culture around latrine use dictated if girls felt comfortable to change absorbents there. Those using AFRIpads may have been able to change faster, as indicated in quantitative findings, [17] facilitating some girls to change at school in those conditions. Still, many girls using AFRIpads or cloths returned home to change. The amount of cloth girls had available to use as absorbent dictated changing practices for some. Girls with limited supply were forced to return home to wash absorbents immediately so they would be available again.

None of the schools provided soap or water basins fit for purpose (even if water was available at a reasonable distance). Most girls washed their absorbents at home and felt comfortable doing so. Girls had more concerns about drying. Interviews revealed that when girls dried absorbents outside they covered them with another cloth. This may explain the unexpected quantitative findings that those drying menstrual absorbents (cloths or reusable pads) outside experienced poorer health (discharge and odour) outcomes [18].

More research is needed on the relationships between irritation, infection, and menstrual management [19, 20]. There were no clear variations across the conditions. Some girls reported burning sensations on extended AFRIpad use, which may have reflected an interaction with labia elongation or hygiene. This may also have been symptomatic of a reproductive-tract or sexually transmitted infection. Girls attributed any irritation to wearing an absorbent for too long, they did not tell others about it or seek advice. Girls’ lack of understanding around vaginal discharge and health may be one source of the poor consistency between self-reported symptoms and laboratory-confirmed reproductive tract infections [21].

Almost all girls experienced menstrual pain and identified this as a barrier to attendance and engagement in the classroom. While girls in this study seemed aware of medication as a treatment for dysmenorrhea, Crichton et al. [22] found that girls in Nairobi were unaware pharmaceutical options existed. Pain and girls’ anxiety about menstrual differences or menstrual disorders have received little attention in interventions to date and recommendations for addressing these are presented below.

In contrast to some past studies (e.g., [3, 16, 23]), we found that most girls were aware of menstruation prior to menarche. This may have been due to the widespread practice of labia elongation and/or the presence of the trial. However, findings indicate that girls were under-informed about practical management and individual variations in menstruation. The education intervention addressed some of this deficit, and provided an opportunity for girls to ask questions and initiate conversations with friends and other support sources. As a result, those in schools receiving the education intervention were more at ease discussing menstruation. Nevertheless, the education fell short in empowering girls to feel in control of their menstrual management. Embarrassment and anxiety around menstruation was evident in girls’ willingness to report others’ struggles with menstruation and soiled garments, rather than their own. A similar finding was noted by Mason and colleagues [3], and did not appear to be impacted by the intervention. While studies have suggested that vaginal practices such as labia elongation may be a source of agency, [24] this did not appear to be the case in this study where social pressure and fear pervaded. Unclear and conflicting information about the practice was a source of anxiety among the girls.

Support from peers, teachers and family were integral to girls’ experience. The impact of the interventions, and particularly the education, on girls’ ability to support each other during menstruation may have been an important, unmeasured variable driving some of the improvements identified. It is unclear if teacher support, such as providing a safe-place for girls to change in their own homes, keeping spare skirts at school for girls, or changing the colour of the school uniforms, were responses to the intervention or actions teachers would have taken independently. However, it is likely that the trial and the involvement of the partner NGO in the school drew attention to this issue.

### Trial implementation

Interviews confirmed implementation issues reported in the trial outcomes paper [11]. If girls were absent during education or pad provision they missed receiving these interventions. Girls shared information from education sessions among themselves, and this study suggests education may have led to greater openness among peers. Thus other girls in the education intervention schools likely indirectly benefitted from the intervention, even if they were absent on the day of the education sessions. The finding that teachers in non-education schools provided some menstrual information is important in understanding the trial results. While this reflects real-world practice, it is possible the provision of pads in pad-only schools prompted greater attention to this education. In a similar way, the inclusion of instructions on making pads from cloth, combined with the fact that this was undertaken in a group and distributed at one of the two education schools, may all contribute to the lack of variation in effects across the interventions in the trial. The inclusion of mediation analyses in future trials would be able to further elucidate pathways of effect. Qualitative findings presented here are consistent with the null findings of the impact of the intervention on shame and insecurity outcomes, with girls across conditions expressing anxiety.

### Strengths and limitations

As a follow-up to a trial, this study was able to provide a comparison of experiences following different interventions. Schools were quasi-randomised to conditions and thus interventions were independent of participant characteristics. Participants were purposively sampled to include a range of classes and ages, however, girls who had dropped out of school or were absent on the day interviewers came to the different schools were unable to be selected for interview. The sample size was limited by available resources and time constraints as interviews were undertaken concurrent with survey data collection. The small sample size remains a limitation of this study. NGO staff assisted in translation and selection of participants and may have also introduced bias. Interview transcription included English translations provided verbally during the interviews, in addition to girls’ responses translated by different interpreters during transcription. This provided a double-translation of interviews, ensuring accuracy. There were insufficient funds for back-translation efforts, and four interviews had only English transcriptions due to funding limitations. Interviews were subject to self-report biases. This was evident in girls’ greater ease in reporting others’ difficulties with menstruation rather than their own. The presence of a foreign interviewer may have meant further hesitance to share openly about menstrual experiences. However, translation provided by local, female research assistants from the partner NGO facilitated rapport with participants.

### Implications for research

Interviews highlighted measurement issues in menstrual health research. For school attendance, frequent part-day absences for changing absorbents are missed by most measures which record attendance once per day. Girls did not consider these absences as ‘*missing school’*, so studies reliant on self-reported absences may be biased. Interviews indicated girls used soap very sparingly. The amount of soap used may have implications for the accuracy of menstrual hygiene estimates that rely on yes/no responses to soap use (e.g., [18]). In evaluating menstrual hygiene, current items fail to capture use of soap alternatives, such as the plant substitute report in this study. Further, findings indicate that items capturing drying practices have been too simplistic. These are likely to be insufficient if they only collect information about location but not if absorbents are covered with another cloth. For future studies of menstrual health, questionnaire items asking simply if girls knew about menstruation prior to menarche are insufficient and misleading where girls have substantial deficits in practical knowledge. We recommended that future studies evaluate the reliability and validity of more detailed questions. Asking girls about their most recent menses, rather than what they *usually* do or how they *usually* feel during menstruation may improve the accuracy of self-reports. It may also be useful to ask about others’ menstrual practices and compare these with self-reports to identify bias in studies assessing new measures.

Interviews revealed hitherto overlooked items in studies of menstrual experience. These include the way girls record their cycle, with interviews finding girls were often caught unprepared on the first day of their menses. Teachers and peers were important contributors to girls’ experiences. Greater attention to these sources of support in studies of school attendance and menstrual experience are needed. The broader impact of interventions, such as personal or family funds saved by the provision of absorbents in interventions, or improvements in girls’ feelings of control over their own bodies are outcomes that have not yet been assessed in intervention studies. Future trials should be of sufficient size, and employ adequate quantitative survey assessment to undertake mediation analyses comparing pathways of effect. This would be invaluable in extending menstrual health theory and improving interventions.

### Implications for practice

Results of this study provide a range of recommendations for intervention development. As noted above, the study supported the effectiveness and acceptability of providing reusable cloth pads or puberty education. Beyond increasing access to sanitary products, much of the emphasis in this field has moved towards providing water, sanitation and hygiene (WASH) facilities in schools (e.g., [25]). In designing these interventions, it should be noted that girls in this study expressed comfort washing their menstrual absorbents at home. They had access to a personal basin, female-only spaces, and often soap. Some girls reported more variable access to soap, or using it very sparingly. Improving access to soap may support hygiene practices for girls using cloth or reusable sanitary pads. Results of this study suggest that improved facilities at school may be essential to ensure girls can comfortably change their menstrual absorbents at school, reducing part-day absences. Interventions providing or encouraging use of discrete plastic bags to transport spare absorbents to school and used absorbents home may be of use where there are existing changing facilities, although the acceptability of this strategy to girls should be evaluated. Drying presented a challenge to girls’ feelings of privacy in this study. Recommendations for menstrual hygiene include drying absorbents outside to benefit from air-flow and the antimicrobial properties of sunlight. However, if this recommendation is implemented with other material put over the top of the absorbent, drying times may be increased, increasing anxiety about discovery, and benefits of direct sunlight lost.

Future education interventions should be improved. In this study, girls’ feelings of control over their menstrual management and body remained low. Girls suffered from a lack of information about vaginal discharge, reproductive tract infections, and menstrual irregularities. Future interventions may be improved by providing more detailed practical information about menstruation and menstrual management, educating support people, and emphasising girls’ attention to their own body and experience. Girls were often caught unprepared on the first day of their menses. Encouraging girls to attend to their cycle and bodily cues signalling menses, as well as storing spare absorbents for the first day, may address this issue in the future. Dark coloured school uniforms as implemented in one school may be a way to improve experience and reduce fears of soiling. This study suggests that support from teachers and peers were integral to intervention effectiveness. Education facilitating this communication, such as group exercises during sessions, may therefore be more effective than individual or lecture-based education. Empowering teachers to better inform and support girls by providing education, practical tips and tools for them may boost intervention effectiveness and longevity.

Menstrual pain remains a challenge worldwide. Providing pain relief medications to girls in LMICs would be ethically challenging and difficult to implement without the risk of harm. However, findings from this study hold some alternatives. Many girls did not ask for pain killers, and those that did ask often did not reveal the source of their pain. In some cases, this resulted in the provision (and wasted funds) of the wrong medication. Some girls could report that paracetamol was the drug given. The most recent review has found that non-steroidal anti-inflammatory drugs (NSAID, e.g., Ibuprofen) are effective for the relief of menstrual cramps, and significantly more effective than paracetamol [26]. Future interventions could be improved by empowering girls to report the source of pain and providing education about the best types of medication. Few girls used other strategies, such as stretching or heat, which could also be suggested in education [27]. Finally, interventions which reach parents and caregivers and reduce menstrual stigma more broadly may be important in opening channels of communication so girls can openly talk about their needs.

## Conclusions

In sum, this qualitative work has enhanced understanding of the change process and implementation of the *Menstruation and the Cycle of Poverty* trial. Further, findings have implications for the future development and evaluation of menstrual health interventions. Results are consistent with the quantitative findings from the trial in suggesting that both education and pads were effective interventions to address some menstrual needs and reduce menstrual-related absenteeism. The provision of sanitary pads reduced fears of outer garment soiling, although did not address other menstrual hygiene challenges such as changing and drying the reusable pads. Education was successful in improving understanding and comfort with menstruation, but more practical information was needed. Social support from peers and teachers was an important driver of effects across both conditions. Implications for research and practice have been highlighted.

## Abbreviations

CI: Confidence interval
LMICs: Low and middle income countries
SD: Standard deviation
